# Reconsidering Early HIV Treatment and Supervised Treatment Interruptions

**DOI:** 10.1371/journal.pmed.0010041

**Published:** 2004-11-30

**Authors:** Richard A Koup

## Abstract

Another study casts doubt on the value of early treatment and treatment interruptions. What are the implications of this study for our understanding of HIV pathogenesis, treatment, and vaccine development?

The devastating effects of HIV infection worldwide are reason enough for AIDS researchers to grasp at thin rays of hope. But seldom has a single anecdotal case stimulated as much hope as the 1999 report of an acutely infected patient who appeared to control HIV replication after two short treatment interruptions [1]. This report generated the hypothesis that early antiretroviral treatment (during or very soon after symptomatic seroconversion) allows the incompletely damaged immune system to recover and respond appropriately to virus antigens during treatment interruptions. This, in turn, according to the hypothesis, leads to control of viral replication by a healed and appropriately stimulated immune response to the patient's HIV infection.

Consistent with this hypothesis was the prior finding that early antiretroviral therapy led to induction of HIV-specific proliferative responses similar to those that had been observed in patients with long-term, non-progressing HIV [2]. This led Rosenberg and colleagues to ask whether HIV-specific proliferative responses were a necessary and sufficient cause of long-term non-progression or just an immunologic consequence of controlled virus replication. Their report of virologic control in patients who interrupted therapy after early treatment raised hope that if HIV infection was treated early enough, the immune system could be repaired sufficiently to allow for long-term immunologic control of HIV replication [3]. Unfortunately, that's where the good news ends.

## Enthusiasm Fades

A series of discoveries from clinical trials began to chip away at the enthusiasm for both early treatment of HIV infection and supervised treatment interruptions (STIs) as a way to boost the immune response.

Several small trials of STIs in chronically infected patients were carried out [4], buoyed by the reasonable desire of patients for respite from the unpleasant side effects of the drugs. These trials gave disappointing results, up to and including the emergence of antiretroviral drug resistance in patients randomized to receive STIs. HIV-specific immune responses did increase off therapy, but so did viral loads. The so-called immune boosting probably reflected an immune response to greater viral antigen load but did not represent constructive immune enhancement.

Larger trials clearly showed that STIs were of little if any benefit in chronic infection and that when therapy was stopped, viral loads invariably returned to pre-treatment levels [5]. Other studies indicated that HIV-specific CD4+ T cells were being preferentially infected, often massively, during treatment interruptions [6], and that proliferative responses were more likely to be a consequence—rather than a cause—of decreased HIV replication [7]. Despite multiple attempts, early reports of an inverse correlation between simple HIV-specific T cell responses and virologic control were not confirmed [8]. Where complex T cell functions did show such a correlation, the data indicated that viral replication was adversely affecting the character of the T cell immune response to HIV, and not the other way around [9]. Thus, no evidence of “immune boosting” during STIs and subsequent viral control in the absence of antiretroviral drugs was ever established. Finally, one of the acutely treated patients within Rosenberg's cohort became superinfected with a second strain of HIV despite excellent control of viral replication and significant recognition of the superinfecting strain by the pre-existing T cell response [10].[Fig pmed-0010041-g001]

**Figure pmed-0010041-g001:**
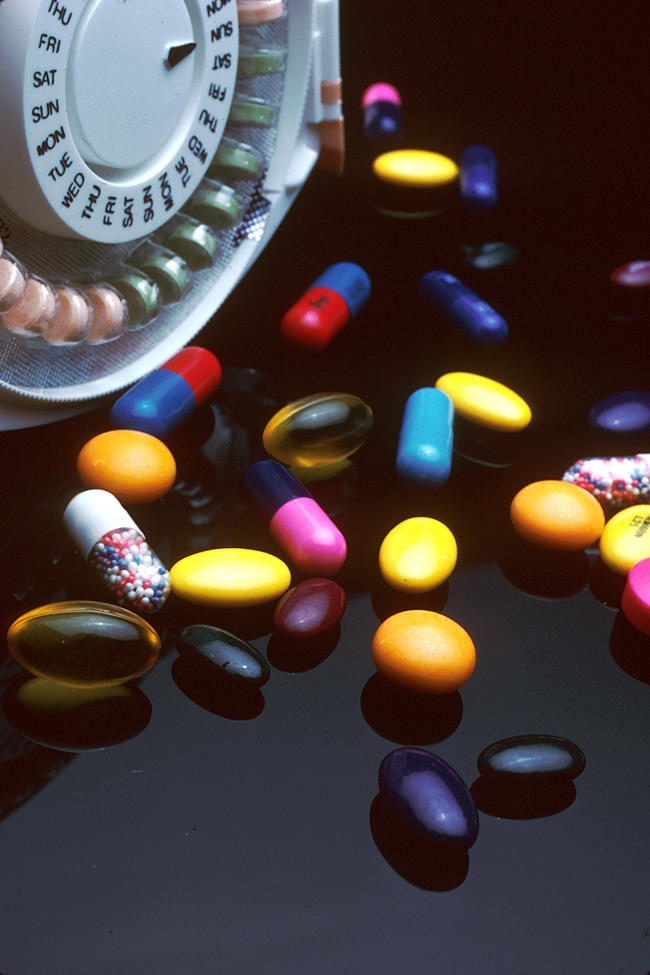
STIs offered patients hope of respite from taking complex regimens, but trials have been disappointing (Photo: J Troha)

## New Findings

Now comes a study in this month's *PLoS Medicine* that found that in 14 patients who were treated early and who had controlled viral loads for at least 90 days, the virologic control was only transient [11]. While one could look at this as a glass half full—these patients achieved a reasonable period of time off antiretroviral therapy—closer scrutiny of the data limits this view.

There was a disconnect between the low viral loads and an unexpectedly high rate of CD4+ T cell decline in several patients. While the small number of patients and the single-arm nature of the study preclude definitive comparisons, it is possible that the early treatment and STIs did not result in a delay in CD4+ T cell decline (and, therefore, initiation of antiretroviral therapy) beyond what would have occurred had the patients received no early treatment.

## Implications of the Study

This study raises important questions in our understanding of HIV pathogenesis, treatment, and vaccine development.

First, why is it that early antiretroviral treatment, even if it does lead to better control of viral replication, does not protect against CD4+ T cell depletion? It is possible that by the time patients present with acute retroviral syndrome their CD4+ T cell reserves (in gut and lymphoid tissues) have been severely depleted, despite the fairly normal CD4+ profile of their peripheral blood. Thus, even low-level viral replication is then sufficient to deplete the remaining central and peripheral reserves [12].

Second, how do these findings affect treatment guidelines during acute infection? None of the current treatment guidelines in either resource-rich or resource-poor settings recommend early antiretroviral therapy. In the light of these new data [11], there does not appear to be a rationale for early antiretroviral therapy in the absence of a clinical trial to assess other interventions in concert with early therapy. The use of therapeutic vaccination is an obvious intervention that still needs to be tested, despite limited efficacy results in treated chronic infection. As such, practice guidelines should continue to caution against early treatment unless associated with a randomized clinical trial.

Finally, is this good or bad news for HIV vaccine development? Since most current vaccine strategies are based upon the hypothesis that induction of T cell immunity will lead to control of viral replication, it is difficult to be optimistic when a strong and broad immune response is unable to prevent disease progression. However, one must recall that phenotypic and functional assessments of HIV-specific T cell responses, even in antiretroviral-treated patients, show that these responses clearly differ from responses against viruses that are normally cleared or controlled by the immune system [9]. Therefore, the T cell responses in the patients treated for acute HIV infection in Kaufmann et al.'s study were induced upon a dramatically altered immune background. It remains to be determined how much this adversely affects the HIV-specific immune response, and whether an immune response generated by vaccination *before* any HIV replication (a prophylactic vaccine) might be better able to control virus replication. Far be it for us to stop grasping at rays of hope.

## Abbreviation

STI: supervised treatment interruption
